# A Systematic Review of Digital Interventions to Improve ART Adherence among Youth Living with HIV in sub-Saharan Africa

**DOI:** 10.1155/2022/9886306

**Published:** 2022-09-26

**Authors:** Kevin Griffee, Roxanne Martin, Ashley Chory, Rachel Vreeman

**Affiliations:** ^1^Icahn School of Medicine at Mount Sinai, New York 10029, NY, USA; ^2^Arnhold Institute for Global Health, Department of Global Health and Health Systems Design, Icahn School of Medicine at Mount Sinai, New York 10029, NY, USA

## Abstract

An estimated 3.4 million youth aged 15–24 years live with human immunodeficiency virus (HIV), a majority of whom reside in sub-Saharan Africa (SSA). Youth living with HIV (YLHIV) generally maintain lower levels of antiretroviral therapy (ART) adherence compared to other age groups, which has negative impacts on long-term clinical outcomes. Given expanding mobile phone and Internet usage among youth in SSA, and a need for strategies to increase ART adherence, this review systematically assessed whether digital interventions could be used to improve YLHIV ART adherence in SSA. PRISMA 2020 guidelines were followed, and PubMed and Scopus databases were searched using terms to reflect the population of interest and different digital intervention strategies to improve ART adherence. Experimental or quasi-experimental studies in SSA evaluating the quantitative effect of digital interventions on YLHIV ART adherence were included. 3849 articles and abstracts, and 122 full texts were screened by two researchers (KG and RM). A third researcher (AC) resolved conflicts. Data were extracted from six eligible articles published between 2017 and 2021. Interventions from included studies lasted 13–96 weeks and took place in Kenya, Nigeria, Uganda, and Zimbabwe. Two of the six studies found significant intervention-related improvements in viral suppression. Of these two studies, one used short message service (SMS) for appointment and daily adherence reminders, and the other combined weekly SMS reminders with phone calls, support groups, home visits, and clinic-based counseling. The four remaining studies, using SMS and Internet-based interventions, did not find any significant adherence-related improvements. This review provides mixed evidence for using digital interventions to improve ART adherence among YLHIV in SSA. Given the relative novelty of using digital interventions in this context, further research is required to evaluate their effectiveness in improving youth ART adherence.

## 1. Introduction

Globally, youth remain a high risk group for human immunodeficiency virus (HIV), with cases attributed to both vertical and horizontal transmission [1]. Overall, two out of every seven new HIV infections worldwide involve young people aged 15–24 years, and most new infections occur in sub-Saharan Africa (SSA) [2]. In 2019, 3.4 million youth were living with HIV, and acquired immunodeficiency syndrome (AIDS) related deaths among young people were estimated to be 46,000 [2]. While antiretroviral therapy (ART) coverage among youth living with HIV (YLHIV) has increased substantially in the last decade [3], significant obstacles to effective HIV care delivery remain [4, 5]. HIV-related stigma, fear of disclosure, lack of social support, limited HIV-related knowledge, a shortage of youth-specific services, and limited ART formulations available to youth remain critical threats to engagement and retention in care [4, 5]. Consequently, YLHIV generally have lower levels of ART adherence compared to other age groups [4], which increases the risk of viral failure, drug resistance, and secondary HIV transmission [6]. Novel approaches to improving ART adherence among youth are therefore needed.

Digital health interventions for remote youth engagement, medication support, and counseling services represent a promising approach to improving ART adherence in this high-priority demographic. Digital health has received increased attention in recent years as Internet and mobile phone usage has expanded substantially worldwide, particularly in low- and middle-income countries [7]. A 2017 Pew Research study examining mobile phone use in six SSA countries–Ghana, Kenya, Nigeria, Senegal, South Africa, and Tanzania–found that most adults owned mobile phones, and a growing percentage owned smartphones with access to applications and the Internet [8].

To our knowledge, there has yet to be a systematic review focused on the specific effect of digital health interventions to promote ART adherence among YLHIV in SSA. Previous reviews of digital interventions to support ART adherence found short message service (SMS) interventions [9–11], as well as Internet-based and combined Internet plus SMS interventions [11], to significantly improve ART adherence among both adolescents and adults living with HIV in countries across Africa, Asia, Europe, North America, Oceania, and South America. Another review found SMS reminders to improve ART adherence among adolescents living with HIV (ALHIV) in five out of seven included studies (from North America, South America, and SSA), but a meta-analysis found no significant effect [12]. Two of these seven studies are also included in the present review [6, 13]. This systematic review aimed to evaluate the current use and effectiveness of digital interventions to improve ART adherence with a particular focus on the population bearing the brunt of HIV-related morbidity and mortality today: YLHIV in SSA.

## 2. Materials and Methods

**Figure 1:**
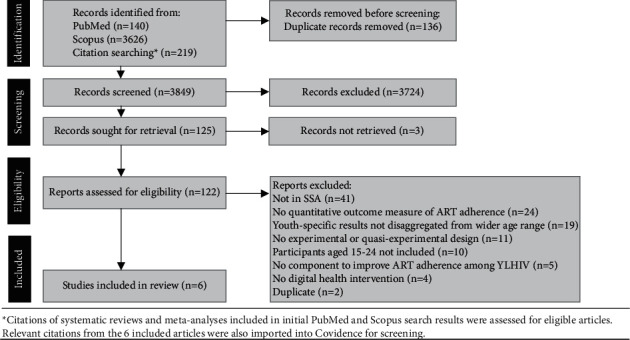
PRISMA study selection flow diagram.

The preferred reporting items for systematic reviews and meta-analyses (PRISMA) 2020 guidelines were followed to conduct this review [14]. The PRISMA study selection flow diagram is shown in Figure 1.

### 2.1. Search Strategy

The literature search was conducted on 30 June 2021 in PubMed and Scopus using the following terms: (“adherence”) AND (“Africa”) AND (“ART” OR “ARV”) AND (“HIV” OR “AIDS”) AND (“adolescen^*∗*^” OR “teen^*∗*^” OR “young adult”) AND (“computer” OR “digital” OR “eHealth” OR “electronic” OR “Facebook” OR “gaming” OR “laptop” OR “mHealth” OR “mobile” OR “phone” OR “SMS” OR “social media” OR “tele^*∗*^” OR “text messag^*∗*^” OR “video” OR “WhatsApp” OR “wireless”). Reference lists of systematic reviews and meta-analyses included in our initial search results were also assessed for eligible articles. Relevant references from studies meeting eligibility criteria were included as well. Citations were downloaded to EndNote (version X9.3.3) citation manager software and subsequently imported into Covidence [15], a systematic review management software.

### 2.2. Eligibility Criteria

Studies were included if they met the following criteria: (1) published in English in an available full text format, (2) implemented in SSA, (3) evaluated an intervention that included at least one component to improve ART adherence among YLHIV, (4) utilized a digital health intervention, defined as health services delivered via digital technologies such as mobile phones or websites [16], (5) included at least one quantitative outcome measure of ART adherence, (6) utilized an experimental or quasi-experimental design, and (7) included youth aged 15–24 years or disaggregated youth-specific results from a wider age range (Figure 1).

### 2.3. Study Selection

Titles and abstracts were screened by two researchers (KG and RM), and conflicts were resolved by AC. The process was repeated for full texts whereby remaining articles were assessed for eligibility by KG and RM, and conflicts were resolved by AC. Studies were excluded if they did not meet the eligibility criteria (Figure 1).

### 2.4. Data Extraction and Analysis

The following data were extracted from included articles and organized in a Microsoft Excel (version 16.54) spreadsheet by KG: title, author, publication date, journal/volume/issue/pages, country, study design, study population, participant ages, sample size, intervention type, intervention length, adherence measurement method, and main adherence outcomes. Results were assessed for statistical significance and compared across studies.

### 2.5. Quality Assessment

Evidence quality was assessed using two methodologies: risk of bias and strength of evidence. The Risk of Bias Tool, developed by the Cochrane Collaboration, provides a process for assessing the risk of bias for randomized controlled trials [17]. The Strength of Evidence Tool, developed by the National Heart, Lung, and Blood Institute, assesses quality for various study types, including pretest-posttest designs [18]. These tools were selected in order to accurately evaluate included articles with different study designs and outcome measures. Two researchers (AC and RM) independently rated each article on risk of bias and strength of evidence; disagreements were settled after discussion and by consensus. Possible outcomes for risk of bias included “low,” “high,” or “unclear.” Possible outcomes for strength of evidence were “good,” “fair,” or “poor.” The Risk of Bias Tool was reserved for randomized controlled trials, and the Strength of Evidence Tool was reserved for pretest-posttest studies. “N/A” was used to denote studies for which the use of a particular tool was not appropriate.

## 3. Results

### 3.1. Literature Search Results

Figure 1 shows the results of the literature search. Searches returned a total of 3985 articles, 136 of which were removed as duplicates. The titles and abstracts of the remaining 3849 articles were screened, and 3724 studies were excluded. Following a full text review, 6 studies met our eligibility criteria and were included in this systematic review (Figure 1).

### 3.2. Study Characteristics

Study characteristics are shown in Table 1. The included studies were all published between 2017 and 2021. Two studies were conducted in Nigeria [4, 6], two in Uganda [13, 20], and one each in Kenya and Zimbabwe [19, 21]. Participants were recruited from local health clinics and were between the ages of 15–24 years [4, 6, 13, 19, 20], except for one study that included participants aged 13–19 years [21]. The latter study was included given the substantial degree of overlap with the 15–24 age range specified in eligibility criteria [21]. The route of HIV infection was not indicated in any of the included studies. Five studies were randomized controlled trials [4, 6, 13, 20, 21], and one study used a pretest-posttest design following a digital intervention [19]. Interventions lasted between 13 and 96 weeks [19, 21], and sample sizes ranged from 90 to 500 participants [19, 21].

### 3.3. Study Interventions and Measures

Table 1 describes the interventions and adherence measures used by the included studies. Three studies exclusively used SMS to provide ART adherence support to study participants [6, 13, 20]. Linnemayr et al. included a weekly SMS reminder (1-way) group and an SMS reminder with response option (2-way) group [13], and MacCarthy et al. included an individual SMS adherence feedback (T1) group and a combined individual plus peer adherence feedback (T2) group [20]. In both studies, adherence was measured via electronic medication monitoring devices and compared to a control group [13, 20]. Abiodun et al. had just one treatment group that received interactive SMS reminders for ART adherence and follow-up appointments, and measured adherence via viral load, self-report, and pill counts [6]. Mavhu et al. utilized a multilevel intervention combining SMS, phone calls, support groups, home visits, and clinic-based counseling, and measured adherence using viral load [21]. In-depth interviews were conducted with participants, healthcare workers, support group leaders, and community adolescent treatment supporters, and were analyzed qualitatively as part of a process evaluation to understand the experiences and support needs of YLHIV [21]. The remaining two studies used Internet-based interventions called ELIMIKA and SMART Connections to promote social support and improve HIV-related knowledge among YLHIV [4, 19]. These studies measured ART adherence, as well as HIV-related knowledge, via self-report [4, 19].

### 3.4. ART Adherence Outcomes

Adherence outcomes are summarized in Table 1. Two studies found statistically significant intervention-related improvements in adherence [6, 21]. The first, by Mavhu et al., was conducted in Zimbabwe and found a decreased prevalence of virological failure or death in the intervention compared to the control group [21]. In the intervention group, 52 (25%) of the 209 adolescents experienced virological failure or had died at 96 weeks, compared to 97 (36%) of the 270 control participants (*p* = 0.03) [21]. Qualitative analysis of participant interviews suggested that the multiple intervention components acted synergistically to improve treatment literacy, build self-esteem, and habituate adherence behavior among participants [21]. The second study, by Abiodun et al., was conducted in Nigeria and found that the intervention group receiving daily interactive SMS adherence reminders had significantly lower mean viral load (*p* = 0.044) and log viral load (*p* = 0.001) compared to the control group at 20 weeks [6]. No statistically significant differences in adherence were found using pill count or self-reported measures [6].

The remaining studies did not report statistically significant adherence-related findings [4, 13, 19, 20]. While MacCarthy et al. found slightly improved adherence in the Treatment 2 (T2) group receiving both individual and peer adherence feedback, this result was not significant [20]. After controlling for baseline adherence, post-intervention adherence in the T2 group was 2.4% higher than in the control group (95% CI −3.0, 7.9) [20]. ART adherence in the Treatment 1 (T1) group receiving only individual adherence feedback was 3.8% lower than the control group (95% CI −9.9, 2.3) [20]. Linnemayr et al. found that ART adherence tended to decrease with the intervention but not significantly [13]. Mean adherence was 67% in the control group, 64% in the 1-way SMS group (*p* = 0.27), and 61% in the 2-way SMS group (*p* = 0.15) [13]. Ivanova et al. found that while participants were satisfied overall with the ELIMIKA digital platform, there were no statistically significant changes in how participants rated perceived importance of maintaining adherence (*p* = 0.75), perceived confidence in maintaining adherence (*p* = 0.58), or intentions to maintain adherence (*p* = 0.50) pre-intervention versus post-intervention [19]. Dulli et al. similarly found that self-reported adherence did not differ significantly between the control and the intervention group (*p* = 0.57) [4]. However, both Ivanova et al. and Dulli et al. did find significant post-intervention improvements in HIV-related knowledge [4, 19].

### 3.5. Quality Assessment

Risk of bias and quality of evidence results are reported in Table 1. Three randomized controlled trials were evaluated to have a “low” risk of bias [6, 13, 21]. In contrast, the risk of bias was “high” in the study by Dulli et al. and “unclear” in the study by MacCarthy et al. [4, 20]. The Risk of Bias Tool did not apply to the pretest-posttest design utilized by Ivanova et al. [19]. Strength of evidence was rated as “fair” in the pretest-posttest study by Ivanova et al. [19]. The Strength of Evidence Tool did not apply to the randomized controlled trials employed in other studies [4, 6, 13, 20, 21].

## 4. Discussion

This systematic review examined the effect of digital intervention strategies on ART adherence among YLHIV in SSA. Overall, two studies found statistically significant intervention-related improvements in ART adherence, and four studies did not. All studies were published relatively recently, demonstrating the novelty of using digital adherence interventions for YLHIV in SSA and the need for future research in this area. While it is difficult to make conclusions based on the limited number of included studies, a few observations are worth noting.

Both studies with statistically significant findings used viral load-based adherence measures [6, 21], considered the “gold standard” for monitoring HIV treatment [22]. Other studies used electronic or self-reported measures [4, 13, 19, 20], which some articles have described as providing less accurate estimates of adherence [22–25]. Dulli et al. and Linnemayr et al. discuss the lack of a viral load measure to be a limitation of their study designs [4, 13]. Further, Abiodun et al. attribute the lack of a correlation between viral load and pill count, visual analog scale (VAS), or AIDS Clinical Trials Group (ACTG) questionnaire results, to the shortcomings of the latter three measures, including the frequent accumulation of leftover pills and the effects of forgetfulness and social desirability on self-reported information [6]. Future studies should consider using viral load in order to most accurately measure the effect of digital interventions on ART adherence.

Of the four studies utilizing SMS in their interventions, two found statistically significant improvements in ART adherence [6, 21], and one study found a nonsignificant improvement in adherence [20]. In comparison, studies using Internet-based interventions did not report significant adherence-related findings [4, 19]. This may suggest that SMS-based adherence interventions have relatively more impact in these SSA settings, where basic mobile phones are generally more available than smartphones, and where text messaging is generally more pervasive than social media- or Internet-use [8]. These findings are consistent with a 2017 systematic review that examined the perceived feasibility of various digital platforms and observed that mHealth (SMS and phone call) interventions were rated as highly feasible in 75 percent of studies, whereas eHealth (Internet-based) interventions were highly feasible in just 45 percent of studies [11].

The included studies with statistically significant findings also trialed these interventions among relatively younger participants, with Mavhu et al. studying youth aged 13–19 years and Abiodun et al. studying those aged 15–19 years [6, 21]. Other studies used broader age ranges that included youth over the age of 19 [4, 13, 19, 20]. A 2018 systematic review found caregiver support to be a key facilitator of ART adherence among ALHIV in SSA [26]. Caregiver support may play a greater role in health care maintenance for younger youth compared to older youth [21, 27] and may have therefore potentiated the effect of digital interventions on ART adherence in younger participants. This may also help explain why the impact of the intervention on viral load was more pronounced in 13–16 year-olds than in 17–19 year-olds in the study by Mavhu et al. [21]. However, further investigation of this possible explanation is needed.

### 4.1. Limitations

This review included only six studies, therefore limiting our ability to make conclusions regarding the efficacy of digital interventions to improve ART adherence. Furthermore, included studies represented only four countries in SSA and utilized a limited array of digital interventions, therefore making it difficult to generalize our findings to all of SSA and all digital intervention types. The substantial variability in some study characteristics, including sample size, intervention length, and adherence measurement, also made it challenging to compare across included studies. However, each of the included studies used rigorous methods, with three out of five randomized controlled trials having a “low” risk of bias and the one pretest-posttest having a “fair” strength of evidence. Moreover, the recency of each study's publication adds greater relevancy to its findings as the digital health landscape continues to evolve.

## 5. Conclusions

This review provides mixed but promising evidence for using digital interventions to improve ART adherence among YLHIV in SSA. Mobile phone and Internet usage in SSA is still not universal and will likely continue to grow in the coming years if historical trends are any indication [8]. With this growth may come greater acceptance and effectiveness of digital interventions to improve ART adherence among YLHIV. Rigorous implementation research studies that evaluate how to most effectively and sustainably use digital interventions for YLHIV adherence are urgently needed in these settings.

## Figures and Tables

**Table 1:** Characteristics of included studies.

Author, year	Country	Study design	Sample size (N)	Age range (years)	Intervention type	Intervention length (weeks)	Adherence measurement method	Main adherence outcomes	Risk of bias	Strength of evidence
Linnemayr et al. 2017 [13]	Uganda	RCT	332	15–22	Weekly SMS (1-way) and weekly SMS with response option (2-way)	48	Medication event monitoring system (MEMS)	1-Way SMS: 95% CI 0.77–1.14, *p* = 0.27 2-way SMS: 95% CI 0.75–1.12, *p* = 0.15	Low	N/A

Ivanova et al. 2019 [19]	Kenya	Pretest-posttest	90	15–24	ELIMIKA digital platform supporting blog posts, discussion, health care provider Q&A, stories contest, and private messaging	13	Perceived importance, confidence, and intentions associated with maintaining adherence pre-intervention versus post-intervention (multiple items assessed but *p* values for “taking all drugs as prescribed by doctor” presented here)	Importance: *p* = 0.75 Confidence: *p* = 0.58 Intentions: *p* = 0.50	N/A	Fair

MacCarthy et al. 2020 [20]	Uganda	RCT	179	15–24	Weekly SMS with individual adherence level from the previous week (T1) and weekly SMS with individual plus peer adherence levels from the previous week (T2)	36	Wisepill smartpill box device	T1 95% CI: -9.9, 2.3 T2 95% CI: -3.0, 7.9	Unclear	N/A

Mavhu et al. 2020 [21]	Zimbabwe	RCT	500	13–19	SMS reminders, phone calls, monthly support groups, home visits, and clinic-based counseling	96	VL (virological failure defined by a VL ≥ 1000 copies/*μ*L)	Adjusted prevalence ratio: 0.58 (95% CI 0.36–0.94, *p* = 0.03)	Low	N/A

Dulli et al. 2020 [4]	Nigeria	RCT	349	15–24	SMART Connections intervention utilizing private Facebook groups to improve social support and HIV-related knowledge	22	Self-report (ACTG) at 6–9 months post-enrollment	*p* = 0.57	High	N/A

Abiodun et al. 2021 [6]	Nigeria	RCT	212	15–19	SMS reminders for follow-up appointments 48 and 24 hours before the visit date and daily interactive ART adherence SMS reminders requiring participants to respond with “1” if they found the reminder acceptable or “2” if not	20	VL, pill counts, self-report (VAS, ACTG)	VL: *p* = 0.044 logVL: *p* = 0.001	Low	N/A

RCT, randomized controlled trial; VL, viral load; ACTG, AIDS Clinical Trials Group adherence questionnaire; VAS, Visual Analog Scale; T1, treatment 1; T2, treatment 2.

## Data Availability

The data used to support the findings of this review are included within the article and referenced studies.
